# ^1^H-NMR spectroscopy for human 3D neural stem cell cultures metabolic profiling

**DOI:** 10.1186/1753-6561-7-S6-O8

**Published:** 2013-12-04

**Authors:** Daniel Simão, Catarina Pinto, Ana P Teixeira, Paula M Alves, Catarina Brito

**Affiliations:** 1 iBET, Instituto de Biologia Experimental e Tecnológica, 2780-901 Oeiras, Portugal; 2 Instituto de Tecnologia Química e Biológica, Universidade Nova de Lisboa, 2780-157 Oeiras, Portugal

## Background

The current lack of predictable central nervous system (CNS) models in pharmaceutical industry early stage development strongly contributes for the high attrition rates registered for new therapeutics [1]. Thus, there is an increasing need for a paradigm shift towards more human relevant cell models, which can closely recapitulate the *in vivo *cell-cell interactions, presenting higher physiological relevance by bridging the gap between animal models and human clinical trials. In this context, human 3D *in vitro *models are promising tools with great potential for pre-clinical research, as they can mimic some of the main features of tissues, such as cell-cell and cell-extracellular matrix (ECM) interactions [2,3]. Moreover these complex cell models are suitable for high-throughput screening (HTS) platforms, essential in drug discovery pipelines by reducing both costs and time in clinical trials [2,4]. However, despite important advances in the last years and the increasing clinical and biological relevance, the full establishment of human 3D *in vitro *models in pre-clinical research requires a significant increase in the power of the available analytical methodologies towards more robust and comprehensive readouts [4]. With the emergence of systems biology field and several "-omics" technologies, such as metabolomics, it became possible to have a more mechanistic approach in the understanding of cellular programs. ^1^H-nuclear magnetic resonance (^1^H-NMR) spectroscopy is a powerful and widely accepted high resolution methodology for a number of applications, including metabolic profiling [5]. Despite the low sensitivity when compared with mass spectrometry (MS), ^1^H-NMR profiling presents several advantages, enabling a non-invasive and non-destructive quantitative analysis requiring only minimal sample preparation [5].

In this work we present the development of a robust and optimized workflow for the exometabolome profiling of 3D *in vitro *cultures of human midbrain-derived neural progenitor cells (hmNPC).

## Materials and methods

### Cell culture

hmNPC were isolated and routinely propagated in static conditions, on poly-L-ornithine-fibronectin (PLOF) coated plates, in serum-free expansion medium, containing basic fibroblast growth factor and epidermal growth factor, as previously reported [6]. hmNSC were cultured in stirred systems as neurospheres for 7 days, with a 50% media changes every at day 3 [7]. All experiments were performed in 500 mL shake flasks (80 mL working volume), with orbital shaking at 100 rpm. Cultures were maintained at 37°C, in 3% O_2 _and 5% CO_2_.

### Sample Preparation

Neurospheres harvested at day 7 were plated on PLOF-coated plates. A washing step with PBS was performed before adding fresh medium (Neurobasal medium (Invitrogen) supplemented with 2% of B27, 2 mM of Glutamax (Invitrogen), 100 μM dibutyryl c-AMP (Sigma-Aldrich), and 10 μg/mL gentamycin (Invitrogen)) to the culture. Samples of supernatant were then collected at 6, 12, 24 and 48 hours after media exchange and stored at -20°C. Neurospheres were harvested and total protein was quantified with Micro BCA Protein Assay Kit (Pierce), according to manufacturer's instructions. Prior to NMR analysis, samples were thawed and filtered using Vivaspin 500 columns (Sigma-Aldrich) at 14,000x*g*, in order to remove high molecular weight proteins and lipids that induce baseline distortions and peak broadening due to protein binding. To minimize variations in pH, 400 μL of filtered samples were mixed with 200 μL of phosphate buffer (50 mM, pH 7.4) with 5 mM DSS-d_6 _[8].

### ^1^H-NMR spectra acquisition and profiling

For NMR analysis, 500 μL of the resulting supernatants were placed into 5 mm NMR tubes. All ^1^H-NMR spectra were recorded at 25°C on a Bruker Avance II+ 500 MHz NMR spectrometer. One-dimensional (1D) spectra were recorded using a NOESY-based pulse sequence (4 s acquisition time, 1 s relaxation time and 100 ms mixing time). Typically, 256 scans were collected for each spectrum. All spectra were phase and baseline corrected automatically, with fine adjustments performed manually. Spectra analysis was performed using Chenomx NMR Suite 7.1, using DSS-d_6 _as internal standard for quantification of metabolites.

## Results

**Figure 1:**
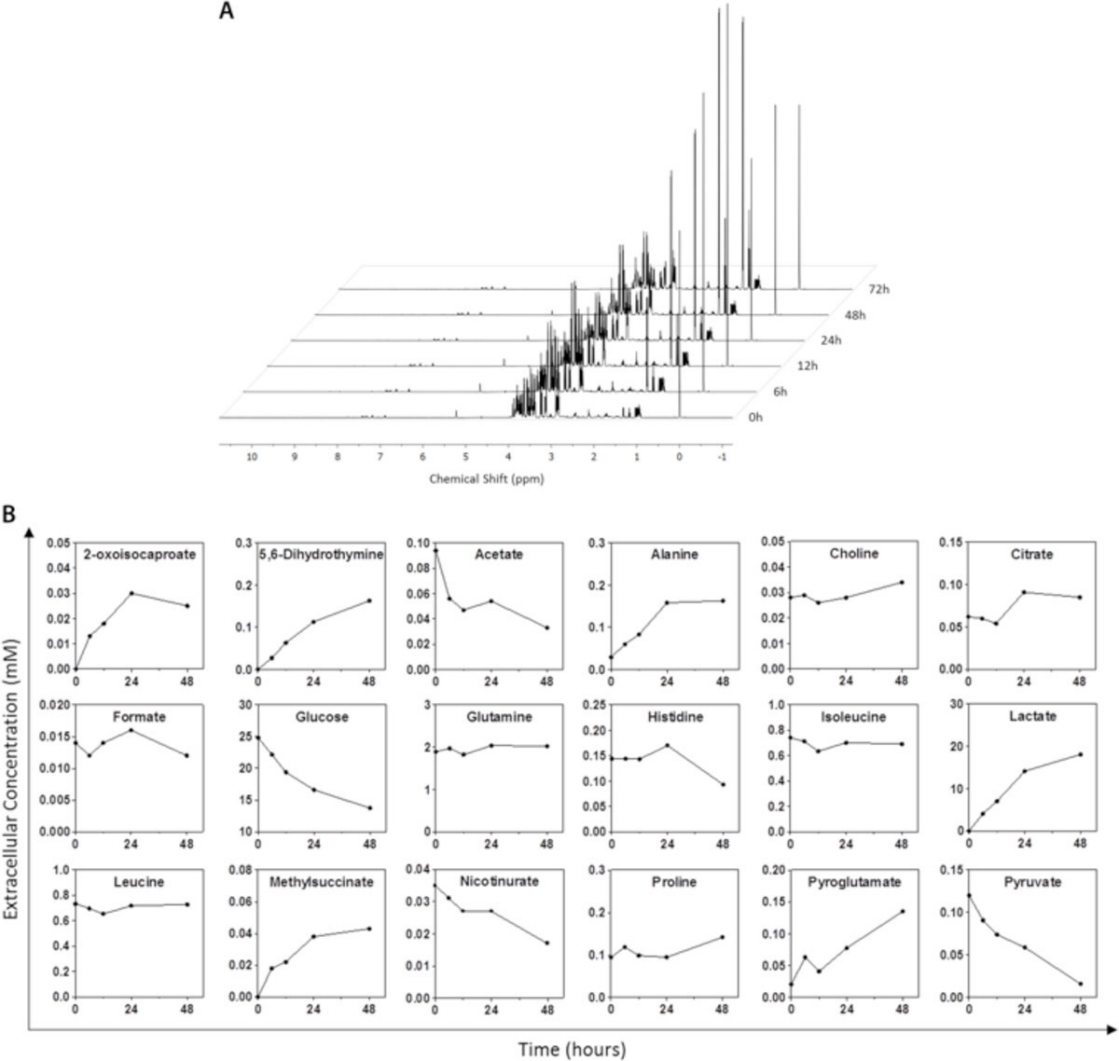
**Typical ^1^H-NMR spectra for hmNPC culture at different time points (A)**. Concentration profiles of the main metabolites quantified in the exometabolome of hmNPC cultures that have significantly changed during 48h of culture (B).

The approach applied in this study for metabolic profiling of the hmNPC cultures using ^1^H-NMR enables an accurate screening of a wide range of metabolites in the extracellular environment (Figure 1A), including amino acids, glucose, lactate, among other substrates and by-products.

Metabolism plasticity has been widely described as closely related with cell pluri/multipotency and cell fate. Stemness programs and cell identity determination are driven mainly by genetic and epigenetic switches, which can modulate cell metabolism, among other cell fate pathways [9]. Thus, the transition from pluri/multipotency towards somatic cell lineages is accompanied by significant metabolic shifts, mainly at energy metabolism levels. In this context, the metabolic study of in vitro cultures of stem cells may contribute with valuable knowledge for the mechanistic understanding of stemness and differentiation pathways.

Our results showed that the hmNPC in an undifferentiated state presented a highly glycolytic metabolism, with high glucose consumption and lactate production rates (Figure 1B), in agreement with previous reports for murine NPC [10]. The profiles observed for glucose consumption and lactate synthesis suggest an almost complete conversion of pyruvate, generated as the final product of glycolysis, to lactate. One key culture parameter that can greatly contribute for a low oxidative metabolism is the fact that neural stem/progenitor cells are typically cultured under physiological low oxygen tension environments. Hypoxic conditions have been widely described as critical for maintaining cell viability and self-renewal, while promoting proliferation and influencing cell fate during differentiation [11]. Moreover, the consumption and depletion of pyruvate present in culture media may suggest not only its conversion to lactate, but may also contribute for the observed alanine synthesis.

Interestingly, even though glutamate could not be detected at significant levels, an accumulation of pyroglutamate was observed, which can be found as N-terminal modification in many neuronal peptides, including pathological accumulating peptides as β-amyloid in Alzheimer's disease. As a free metabolite pyroglutamate can derive both from degradation of proteins containing N-terminal residues or from glutamate/glutamine cyclization. Although it is still a matter of debate, pyroglutamate may act as a reservoir of neural glutamate, which is the main excitatory neurotransmitter in CNS and in high levels becomes a major neurotoxicant [12].

Concerning branched-chain amino acids (BCAA) metabolism it was possible to observe the extracellular accumulation of 2-oxoisocaproate and methylsuccinate as main by-products, although in low rates. In brain metabolism the balance between leucine and 2-oxisocaproate has particular relevance through the establishment of a nitrogen turnover cycle where astroglia cells catabolize leucine into 2-oxoisocaproate, which is then taken up by neurons and converted back into leucine [13,14].

## Conclusions

The methodology presented in this work, enables a straightforward approach for an accurate and reproducible metabolic profiling of multipotent hmNPC 3D cultures. This methodology provides a robust alternative to an array of laborious analytical methods, by taking advantage of the fast and simple sample preparation for NMR spectroscopy and the ease of user-friendly software for spectra profiling, which is often a challenging and time-consuming process due to peak overlapping in complex mixtures such as the mammalian cell culture media. Moreover, this approach can be applied to other multi/pluripotent cell sources, not only for metabolic profiling of *in vitro *cultures but also to study the impact of new therapeutics or toxicants, contributing to generate invaluable data in drug development cascades.
